# 454 pyrosequencing and bioinformatics analysis of the lineage of the broad and potent gp41-directed antibody 10e8

**DOI:** 10.1186/1742-4690-9-S2-O40

**Published:** 2012-09-13

**Authors:** G Ofek, J Zhu, Y Yang, B Zhang, K Mckee, M Louder, J Huang, L Laub, M Connors, J Mascola, PD Kwong

**Affiliations:** 1 Laboratory of Immunoregulation, NIAID, NIH, USA; 2 Vaccine Research Center, NIAID, NIH, Bethesda, MD, USA

## Background

The 10e8 antibody is a novel MPER-specific antibody that is both broad and potent, and lacks detectable autoreactivity. 454 pyrosequencing has recently been shown to allow for a bioinformatics analysis of the genetic record of a broadly neutralizing antibody, once its sequence is known.

## Methods

454 pyrosequencing of B cells from donor N152, from whom 10e8 was isolated, was undertaken to characterize additional variants of 10e8 and to define its lineage. PCR reactions using 10e8 germline-specific heavy and light chain primers were performed and 454 pyrosequencing results analyzed using a multi-step in-house bioinformatics pipeline.

## Results

37,669 IGHV3-15 heavy chain sequences and 91,951 IGLV3-19 light chain sequences were obtained and the distribution of these sequences analyzed by two key parameters – divergence from germline and sequence identity to 10e8. Grid-based clustering allowed for the selection of 61 heavy chain sequences and 48 light chain sequences. These were reconstituted with the appropriate 10e8 light or heavy chain partner, expressed and tested for MPER recognition. 12 heavy chain variants and 27 light chain variants were found to bind MPER, and several of the reconstituted antibodies neutralized a panel of HIV-1 isolates better than the original 10e8. Phylogenetic analyses identified clonally-related sequences, and at least three subgroups of 10e8-like heavy chains were observed. 10e8 light chains meanwhile also showed a few subgroups, though with less sequence variation.

## Conclusion

454 pyrosequencing was thus able to identify clonal variants of 10e8 with substantially improved neutralization potency. Corresponding phylogenetic analyses are providing insight into the ontogeny of 10e8, and structural and biophysical studies are underway to functionally characterize this lineage.

